# Molecular phylogeny and divergence times of *Astragalus* section *Hymenostegis*: An analysis of a rapidly diversifying species group in Fabaceae

**DOI:** 10.1038/s41598-017-14614-3

**Published:** 2017-10-25

**Authors:** Ali Bagheri, Ali Asghar Maassoumi, Mohammad Reza Rahiminejad, Jonathan Brassac, Frank R. Blattner

**Affiliations:** 1 Department of Biology, Faculty of Sciences, University of Isfahan, Isfahan, 81746-73441 Iran; 2 Botany Research Division, Research Institute of Forests and Rangelands, Agricultural Research, Education and Extension Organization (AREEO), 13185–116 Tehran, Iran; 3 Leibniz Institute of Plant Genetics and Crop Plant Research (IPK), 06466 Gatersleben, Germany; 4 German Centre of Integrative Biodiversity Research (iDiv) Halle-Jena-Leipzig, 04103 Leipzig, Germany

## Abstract

The taxa of *Astragalus* section *Hymenostegis* are an important element of mountainous and steppe habitats in Southwest Asia. A phylogenetic hypothesis of sect. *Hymenostegis* has been obtained from nuclear ribosomal DNA internal transcribed spacer (ITS) and plastid *ycf*1 sequences of up to 303 individuals from 106 species, including all 89 taxa currently assigned to sect. *Hymenostegis*, 14 species of other *Astragalus* sections, and two species of *Oxytropis* and one *Biserrula* designated as outgroups. Bayesian phylogenetic inference and parsimony analyses reveal that three species from two other closely related sections group within sect. *Hymenostegis*, making the section paraphyletic. DNA sequence diversity is generally very low among *Hymenostegis* taxa, which is consistent with recent diversification of the section. We estimate that diversification in sect. *Hymenostegis* occurred in the middle to late Pleistocene, with many species arising only during the last one million years, when environmental conditions in the mountain regions of Southwest and Central Asia cycled repeatedly between dry and more humid conditions.

## Introduction

*Astragalus* is with about 2500–3000 species in 250 sections the largest genus of flowering plants^1–7^. It belongs to the legume family (Leguminosae or Fabaceae) that is, after Orchidaceae and Asteraceae, the third largest family of flowering plants^5^ consisting of about 730 genera and nearly 19,300 species. Also its subfamily Papilionoideae, with about 478 genera and 13,800 species^5^, is very species rich. *Astragalus* belongs within this subfamily together with genera like *Oxytropis* and *Colutea* to the Astragalean clade of the so-called *Inverted Repeat Lacking Clade* (IRLC). Its members are all characterized by the loss of one copy of the two 25-kb inverted repeat regions in the chloroplast genome^2,8–10^.

Although species richness seems to be a general characteristic of the legumes, this feature is not evenly distributed among the groups within this family. Thus, Sanderson and Wojciechowski^11^ found that *Astragalus* itself is not significantly more speciose compared to its close relatives, but that species richness is a feature of the entire Astragalean clade in comparison to the other groups of the IRLC. High species numbers can originate through constant accumulation of diversity through time or following a punctuated pattern, where bursts of diversification rates alternate with times of stasis^12^. The latter pattern often results from the evolution of a key innovation that provides the possibility to fill a new ecological niche, or from a key opportunity, i.e. the colonization of a new, formerly not inhabited area and speciation therein^12–15^. For *Astragalus* and the entire Astragalean clade the reason(s) for the high species numbers are still unclear. In an effort to better understand reasons for and timing of species diversification in *Astragalus* we here analyze a large section of the genus, where we put some effort into arriving at a complete species sample.

**Figure 1:**
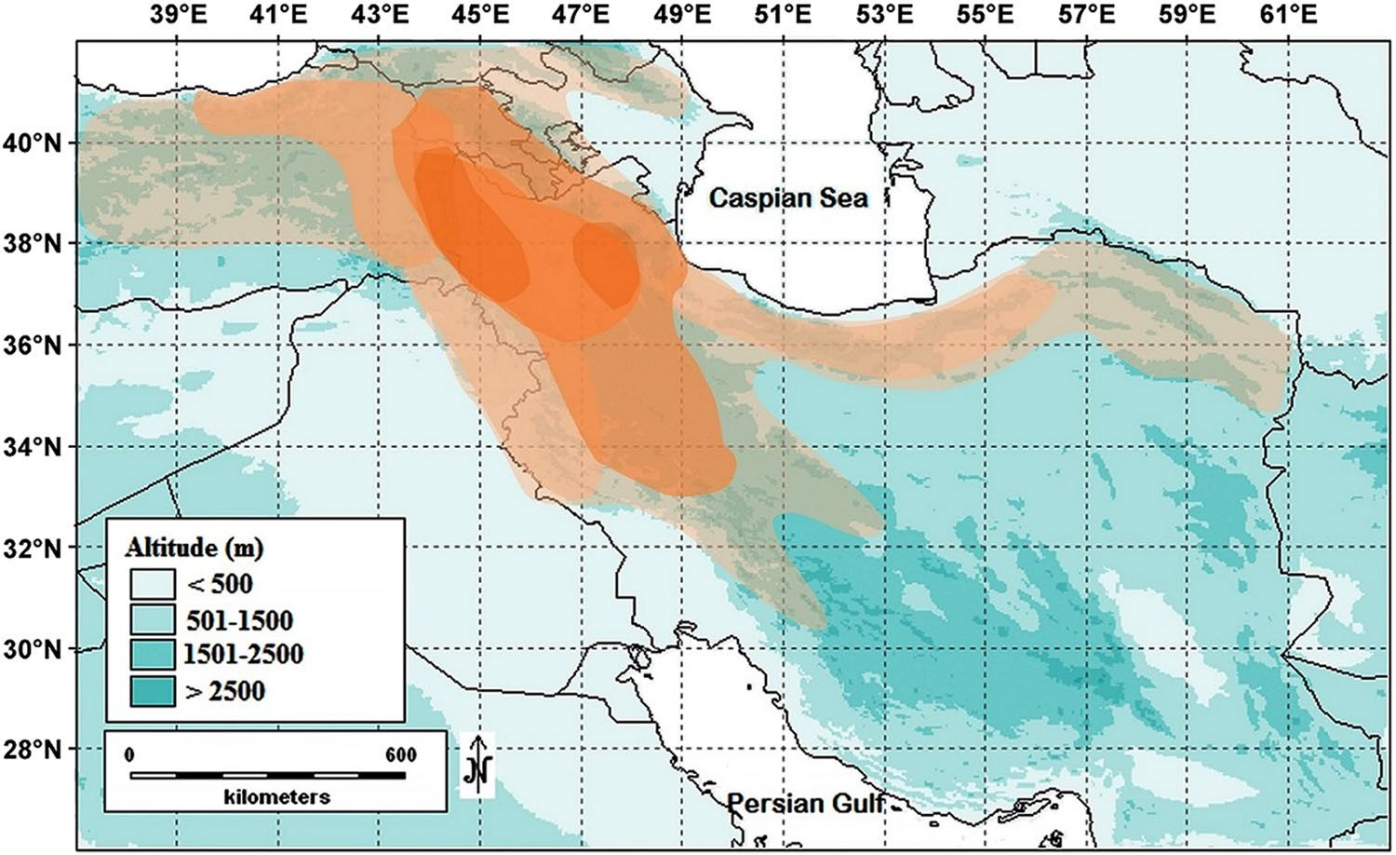
Map of the distribution area of the sect. *Hymenostegis* species. Brown shading indicates differences in species numbers per area from 1–2 (light brown) species to 25–30 species (dark brown)^65^.

*Astragalus* section *Hymenostegis* Bunge was validly published by Bunge^16,17^ including originally 23 species, and was typified with *A*. *hymenostegis* Fisch. & C.A.Mey.^18^. Section *Hymenostegis* has been revised several times^7,19–22^. It is one of the largest sections of Old World *Astragalus*, distributed mainly in Iran and Turkey^23,24^. Of the total number of 89 currently recognized species (Bagheri *et al*., unpublished data), 85 (95.5%) occur in Iran (Fig. 1), from which 71 (79.8%) are endemic to this country^7,23–28^. Members of sect. *Hymenostegis* are morphologically characterized by an inflated calyx, basifixed white hairs, and wide and conspicuous bracts. The section was subdivided by Zarre and Podlech^21^ into two subsections: subsect. *Hymenocoleus* (Bunge) Podlech & Zarre, including only the Turkish endemic *A. vaginans* DC., and subsect. *Hymenostegis*. This division has been based on leaf and stem characters. Podlech and Zarre^7^ followed this treatment in their most recent account of Old World *Astragalus*. Based on an analysis of the nuclear ribosomal DNA internal transcribed spacer (ITS) region of *Astragalus*, sect. *Hymenostegis* (represented by few taxa only) is nested within a clade including tragacanthic *Astragalus* along with other major groups of spiny *Astragalus* sections (including: *Anthylloidei*, *Tricholobus*, *Acidodes*, *Poterion*, *Campylanthus* and *Microphysa*) and seems to be non-monophyletic^29–31^. Up to now no detailed molecular phylogenetic analysis using multiple DNA loci and an exhaustive taxon sampling has been conducted for sect. *Hymenostegis*. Here we describe a study of this taxon based on the nuclear ribosomal DNA (rDNA) internal transcribed spacer region (ITS) and chloroplast *ycf1* sequences for phylogenetic reconstruction, two marker regions which are among the fastest evolving DNA parts found up to now in *Astragalus*. The main purposes of our study are: (*i*) to assess the monophyly of sect. *Hymenostegis*, (*ii*) to examine the evolutionary relationships within this section and to closely related taxa, and (*iii*) to roughly estimate diversification times for the species within the section and put this in the context of the generally high species diversity within the genus and family.

## Results

### Phylogenetic analyses of ITS, *ycf1*, and combined ITS + *ycf1* sequences

#### The alignment of the ITS dataset containing one individual each per species (ITS_106)

Had a length of 621 positions, with 124 variable characters of which 71 were parsimony informative. The parsimony analysis resulted in 359 trees of length 192 with a consistency index (CI) of 0.755 and a retention index (RI) of 0.880. The consensus tree is shown in Supplementary Fig. S1 (all figures indicated by “S” are provided as supplementary materials).

**Figure 2:**
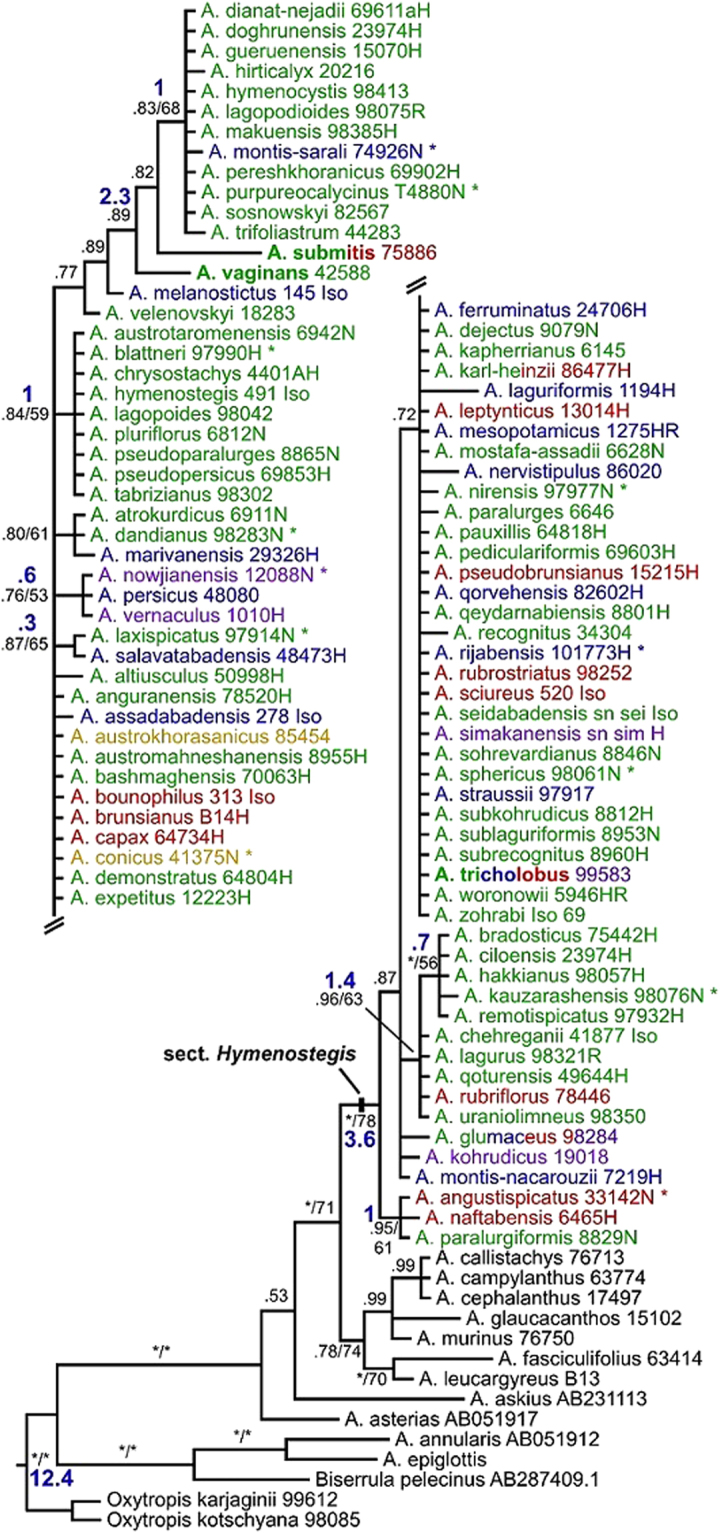
Bayesian phylogenetic tree based on nrDNA ITS sequences and including one individual per sect. *Hymenostegis* species. Clade support values (BI pp/MP bs) are given at the branches of the tree. Asterisks at branches indicate pp ≥ 0.98 and bs ≥ 80%, respectively. Blue numbers provide mean node ages in million years estimated through a Beast analysis. Asterisks behind species names indicate new species names currently in the process of valid publication, while species names given in bold face mark species formerly not included within sect. *Hymenostegis*. Colors of species names refer to distribution areas given in Fig. 3.

The two Bayesian phylogenetic (BI) analyses conducted with this dataset (Nst = 6 *vs*. Nst = 2) resulted in trees with identical topologies and very similar posterior probabilities (pp) for clades. Only one tree is therefore provided (Fig. 2). Also the maximum-likelihood (ML) analysis resulted in very similar species relationships (Supplementay Fig. S2). In these trees the assumed outgroup species *Biserrula pelecinus* groups within *Astragalus* with high support values (see Materials and Methods). Sequence differences among sect. *Hymenostegis* taxa are generally low resulting also in low clade support values. Nine groups, consisting mainly of taxa with identical ITS sequences, are present, the largest with 36 species, smaller clades containing nine, five, four, three and two species sharing their ITS sequence. Three species (*A. submitis*, *A. tricholobus* and *A. vaginans*), assumed to belonging to other sections of the genus, fall inside sect. *Hymenostegis*.

#### The alignment of the ITS sequences for all individuals (ITS_303)

Included 282 individuals from sect. *Hymenostegis* taxa, 19 from other *Astragalus* sections, and two outgroup species. The alignment had a length of 628 positions, of which 506 characters were constant and 85 potentially parsimony informative. The 10,000 most parsimonious trees (max tree limit) had a length of 185 steps (CI = 0.768, RI = 0.948). The ML analysis and BI analyses based on the two different models of DNA sequence evolution resulted in identical tree topologies and support values for the same clades. Therefore, only one tree is provided (Supplementary Fig. S3).

The monophyly of sect. *Hymenostegis* (including the three species mentioned above) is highly supported (pp 1, bs 100%) but taxon resolution within the section is relatively low, i.e. many species share identical ITS sequences, also in cases where these species are morphologically easily discernable. Some small subclades are present within the tree, although they rarely received reasonable clade support values. In this dataset we included between one and six accessions per species, which allows us to infer some cases of non-monophyly for species. Thus, in *A. chrysostachys*, taxa listed as varieties within the species (var. *parisiensis*, var. *dolichourus*, var. *khorasanicus*), group apart from the other *A. chrysostachys* accessions at two different positions in the tree. Varieties *dolichourus* and *khorasanicus* share ITS sequences and morphology with *A. conicus* where they might be taxonomically belonging. In case of var. *parisiensis* probably a new name has to be assigned.

#### The aligned data matrix for the *ycf1*

Data consisted of 1607 characters across 65 accessions including the outgroup species. 1571 characters were constant, and of the 36 variable characters 34 were potentially parsimony informative. The 3648 most parsimonious trees had a length of 183 steps (CI = 0.934, RI = 0.882). Due to the small differences among the *ycf1* sequences, phylogenetic resolution is very low in the MP tree as well as the BI analysis of this dataset. As we obtained for all these individuals also ITS sequences, we do not show these trees but use *ycf1* together with the respective ITS sequences in a combined data matrix.

#### The partition-homogeneity test of ITS and *ycf1* datasets

For the same 65 taxa carried out in Paup* 4.0a150 indicated no major conflicts between both loci. They were therefore combined in a concatenated data matrix for MP, ML and BI analyses.

#### The combined ITS + *ycf1*

**Figure 3:**
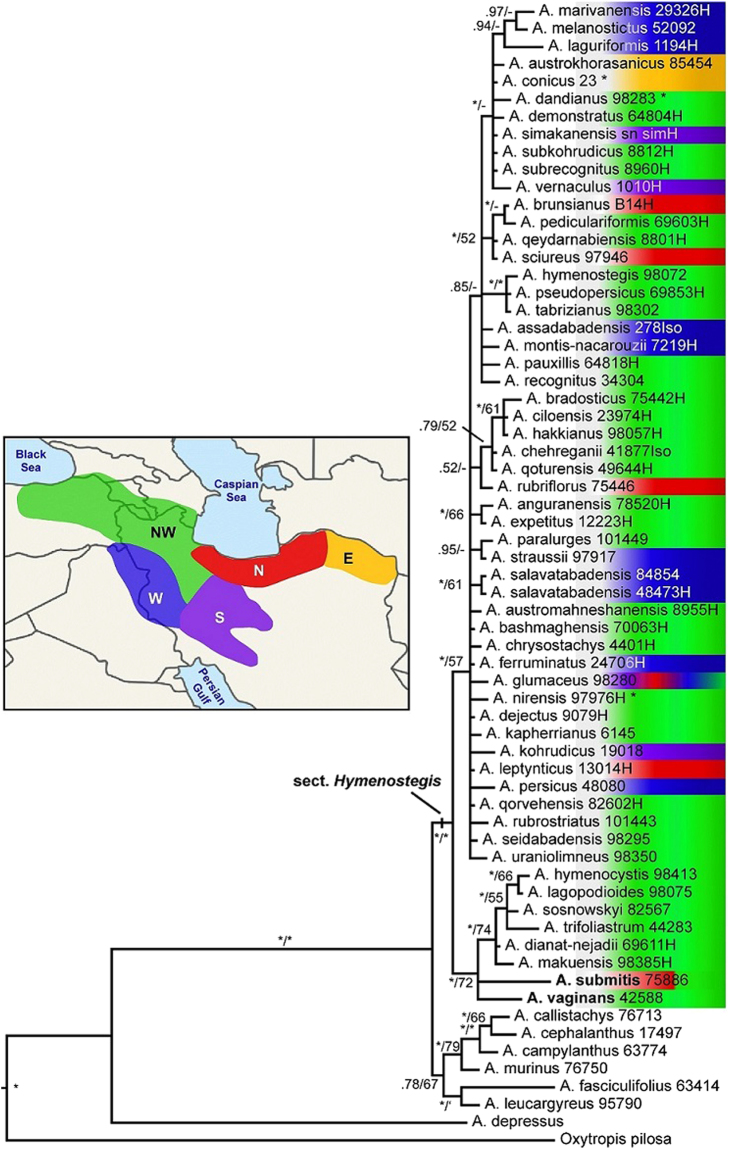
Phylogenetic tree obtained by Bayesian phylogenetic inference of the combined nuclear rDNA ITS and chloroplast *ycf1* datasets of 64 *Astragalus* and one outgroup accession. Clade support values (BI pp/MP bs) are given at the branches of the tree. Asterisks at branches indicate pp ≥ 0.98 and bs ≥ 80%, respectively. Asterisks behind species names indicate new species names currently in the process of valid publication, while species names given in bold face mark species formerly not included within sect. *Hymenostegis*. Regions of origin of the species are color-coded and the areas are provided in the inserted map^65^.

Data matrix consists of 2222 characters across 65 accessions including the outgroup species. 1981 characters were constant, and of the 163 variable characters 78 were potentially parsimony informative. The 216 most parsimonious trees had a length of 305 steps (CI = 0.879, RI = 0.853). BI analysis of the partitioned data resulted in the phylogenetic tree provided as Fig. 3, the ML result is provided as Supplemetary Fig. S4.

Combining both datasets did not result in a substantial increase of phylogenetic resolution in comparison to the ITS dataset, although support values for clades increased. Thus, sect. *Hymenostegis* received maximal support and for several of the smaller species groups within this section reasonable support values were obtained. Also within the combined data several species cannot be discerned based on the sequences of both marker loci.

#### Age estimations for sect. *Hymenostegis*

The MCC tree (Supplementary Fig. S5) resulted in a topology similar to the ITS_106 tree inferred with MrBayes except for the clade consisting of *A. vaginans*, *A. submitis* and 12 other species being sister to all other sect. *Hymenostegis* species. This specific topology is then in accord with the ITS + *ycf1* topology. The analysis inferred a mean substitution rate of 2.44 × 10^−9^ (substitutions/site/year) and resulted in a crown group age of sect. *Hymenostegis* of 3.55 My (95% HPD = 2.13–5.08) with the majority of speciation events being younger than 1.5 My (Supplementary Fig. S5). The ages of the major nodes are given in the phylogenetic tree provided as Fig. 2.

## Discussion

### Monophyly of *Astragalus* and section *Hymenostegis*

In our analyses *Biserrula pelecinus* groups within *Astragalus*, which confirms Wojciechowski’s^32^ suggestion to treat it as *Astragalus pelecinus* (L.) Barneby as part of the genus and regard *Biserrula pelecinus* L. as a synonym of this taxon.

*Astragaalus tricholobus*, *A. submitis* and *A. vaginans* from other closely related sections were placed among the species of sect. *Hymenostegis*. Thus, sect. *Hymenostegis* is paraphyletic when these three species are not included. This finding is consistent with the results of Kazempour Osaloo *et al*. (2003, 2005) and Naderi Safar *et al*.^29–31^. *Astragalus submitis* shares some morphological characters with members of sect. *Hymenostegis*, including the spiny form and inflated calyx at fruiting time. Although, due to its parapinate (not imparipinnate) leaflets and the possession of black and white (not just white) hairs it has been placed in sect. *Anthylloidei* ^7,20–22^. *Astragalus vaginans* shares white hairs (absence of black hairs) and imparipinnate leaflets with the members of sect. *Hymenostegis*, but differs by its elongated stems, long internodes of up to 1.5 cm, and remote leaves^7,21^ from the other sect. *Hymenostegis* species. This species was initially placed in the monotypic section *Hymenocoleus*, which was later reduced to subsection *Hymenocoleus* within sect. *Hymenostegis* ^7,21^. In our analyses it groups together with *A. submitis* plus several other sect. *Hymenostegis* species, providing no clear evidence that would support a separate treatment of this species within a subsection of its own. However, as *A. submitis* and *A. vaginans* exhibit an assemblage of mixed morphological characters and are also, in the context of the otherwise quite uniform ITS and *ycf1* sequences in sect. *Hymenostegis*, relatively different in their DNA characteristics, we speculate that these species could be of hybrid origin. The ITS region did not provide any indications of polymorphic sequence positions in these taxa. This does, however, not exclude reticulate evolution, as the ITS region is prone to fast sequence homogenization^33,34^. Therefore, analyzing nuclear single-copy genes^35,36^ together with a closer evaluation of the morphological characters might be an appropriate way to test this hypothesis.

*Astragalus tricholobus* is nested in the main clade of sect. *Hymenostegis*, sharing its ITS sequence with many other core-*Hymenostegis* species. We could not obtain a *ycf1* sequence for this species, which is the reason that it is lacking from the combined dataset. Morphologically *A. tricholobus* shares many characters like spiny forms, absence of black hairs, imparipinnate leaflets with sect. *Hymenostegis* taxa, although according to Zarre and Podlech (1996), Podlech and Maassoumi (2001) and Podlech and Zarre^7,21,22^ it is assumed to be member of sect. *Campylanthus* or sect. *Tricholobus* ^20,37^. In contrast to the two former species, which show a mixture of typical and atypical characters of sect. *Hymenostegis*, the morphological and molecular characters unambiguously place *A. tricholobus* in sect. *Hymenostegis*.

### Ages and relationships within section *Hymenostegis*

Generally, the DNA sequences analyzed provide relatively few differences for sect. *Hymenostegis* taxa (Figs 2 and 3). However, two major groups can be discerned within the section. The ITS as well as the combined dataset resulted in a clade of species including *A. vaginans*, *A. submitis*, together with additional taxa closely related to *A. dianat-nejadii* and *A. hymenocystis*, with reasonable support values in the latter dataset. This clade is in the Beast analysis and the combined dataset the sistergroup to all other species within the section, while in the ITS trees inferred with MrBayes it forms either a clade within these other species (Fig. 2) or occurs at an unresolved polytomy with other sect. *Hymenostegis* species groups (Supplementary Fig. S2). Due to these inconsistencies and the small genetic differences found, we can draw no conclusions about the internal taxonomic structure of the section from our datasets. The main feature of the group seems to be the very small molecular diversity in sect. *Hymenostegis* in comparison to the morphological well differentiated species belonging to this taxon.

Essentially, there are two reasons for low phylogenetic resolution among species. This could be due to (*i*) the inappropriate choice of molecular marker regions selected. In our study we used, however, with the nrDNA ITS region the fastest evolving universal nuclear marker, and the *ycf1* gene that provided the best resolution in earlier *Astragalus* studies^38^. Therefore, we assume that (*ii*) we deal here with a very rapid radiation, i.e. many nearly simultaneous speciation events resulting in fast changes of morphological characters, which are not equally accompanied by changes in the (neutral) marker regions we used for our study. Here using next-generation sequencing approaches to screen for genome-wide DNA differences seems a more suitable approach to us for resolving such narrow species groups^39–41^. Moreover, these methods could simultaneously identify genome regions contributing to fast morphological changes. However, given the high species numbers within *Astragalus*, the Astragalean clade, Papilionoideae, and the entire Fabaceae, the rapid and young radiation we infer here for sect. *Hymenostegis* is not a phenomenon exclusive to this section but seems to be a general feature of *Astragalus* ^11^ if not for many groups within the entire family^42–44^. Generally, finding an analysis method that could resolve species and species groups within *Astragalus* much better would allow analyzing parallel rapid radiations on a global scale^45–47^ and, thus, infer causes for the species richness observed in the Astragalean clade. For this we want to employ next a RAD-seq analysis^40^ in this section to explore the potential of such methods to obtain higher species resolution in *Astragalus*.

Molecular dating places the separation of sect. *Hymenostegis* from other spiny sections of *Astragalus* into the middle Pliocene (~4 My ago) and the majority of speciation events within the section to the last 1 My. Although these ages should be taken with some caution, as they are resting on a secondary calibration point, and the rather recent crown-group age of section *Hymenostegis* of 3.5 My bases such estimations on only few molecular differences, the low genetic diversity found in both phylogenetic markers supports at least a rather recent diversification of the group. During this time the typical steppe habitats of the sect. *Hymenostegis* species were repeatedly shrinking and expanding due to the Pleistocene climate cycles, resulting in a constant shift between humid times, characterized by expanding forests, and dry periods where the steppes expanded again^48–51^. It was already shown that these climate cycles could drive diversification in plant species through repeated subdivisions of populations resulting in allopatric speciation followed by range expansion when climate conditions became again favorable^44,52–55^. We here hypothesize that this mechanism might have played an important role also in the *Astragalus* species of the Irano-Turanian steppe regions.

Although we used the fastest evolving marker regions known for *Astragalus*, we obtained only rather low resolution within the phylogeny of sect. *Hymenostegis*. This prevents us from providing a reasonable biogeographic scenario for the taxon group. We can only show that the highest species number occurs in the Northwest of the distribution area of the section, i.e. northwestern Iran and eastern Turkey. Species from this area can be found in all clades within the phylogenetic tree (Fig. 3), which could be interpreted as an initial radiation of the sect. *Hymenostegis* in this region followed by multiple dispersals out of this area into neighboring regions. Towards the South, East and West, species diversity sharply drops. This is probably due to the lack of suitable habitats, as towards the southern reaches of Iran the steppe is replaced by dessert vegetation even in the mountains, and in the East and on the Anatolian plateau the environment gets maybe too dry for these *Astagalus* species. However, a formal biogeographic analysis has to await results from genome-wide single-nucleotide polymorphisms that should result in a better resolved phylogenetic tree in comparison to the markers used here.

## Material and Methods

### Taxon sampling

We sampled leaf material for DNA analyses from all species of sect. *Hymenostegis* derived from herbarium vouchers, including most of the type specimens, as well as materials collected during fieldwork. Thus, we checked all relevant collections of the herbaria AKSU, ANK, B, FAR, GAZI, HUI, M, MSB, TARI, W, and WU. All together we included 303 individuals representing 89 species of sect. *Hymenostegis* and 14 species of other sections including ten species from the closely related and spiny sections. These are *A*. *vaginans* (sect. *Hymenocoleus*), *A*. *submitis* (sect. *Anthylloidei*), *A*. *tricholobus* (sect. *Campylanthus*), *A*. *callistachys* (sect. *Microphysa*), *A*. *campylanthus* (sect. *Campylanthus*), *A*. *cephalanthus* (sect. *Microphysa*), *A*. *glaucacanthos* (sect. *Poterion*), *A*. *murinus* (sect. *Anthylloidei*), *A*. *fasciculifolius* (sect. *Poterion*) and *A*. *leucargyreus* (sect. *Adiaspastus*). Another four species represent sections not closely related to the taxon under study. These are *A*. *askius* (sect. *Incani*), *A*. *asterias* (sect. *Sesamei*), *A*. *annularis* (sect. *Annulares*) and *A*. *epiglottis* (sect. *Epiglottis*). Also, two species of *Oxytropis* (*O. karjaginii* and *O. kotschyana*) and *Biserrula pelecinus* were included as outgroup taxa (but see below, 2.4). The herbarium specimens we used were up to 180 years old. Depending on storage conditions of the vouchers they were in diverse states of conservation, resulting also in rather diverse quality of extracted DNA. Therefore, not for all specimens both marker regions could be obtained. We complemented our datasets with sequences downloaded from the GenBank nucleotide database. Finally, we were able to include ITS sequences for 303 individuals representing 89 *Hymenostegis* species (some recognized as new taxa and up to now not formally described) plus 17 non-*Hymenostegis* taxa in our analysis. For the chloroplast *ycf1* dataset we included 65 individuals representing 54 sect. *Hymenostegis* species plus ten outgroup taxa. Taxa, origin and GenBank sequence accession numbers for materials in this study are listed in Supplementary Table S1 (all tables indicated by “S” are available as supplementary materials).

### DNA extraction, amplification and sequencing

Total genomic DNA was extracted from about 10 mg of herbarium or silica-gel dried material with the DNeasy Plant DNA Extraction Kit (Qiagen) according to the instructions of the manufacturer. DNA concentration and quality were afterwards checked on 0.8% agarose gels. The ITS region (ITS1, 5.8 S rDNA, ITS2) was amplified using the primers ITS-A and ITS-B^56^. ITS1 and ITS2 were amplified separately when DNAs from very old herbarium sheets were used; in these cases, the primers ITS-C and ITS-D^56^ together with the primers ITS-A and ITS-B, were used. PCR was performed with 1.5 U Taq DNA Polymerase (Qiagen) in the supplied reaction buffer, 0.2 μM of each dNTP, 50 pmol of each primer, Q-Solution (Qiagen) with a final concentration of 20%, and about 20 ng of total DNA in 50 μl reaction volume in a GeneAmp PCR System 9700 (Perkin–Elmer). Amplification was performed with 3 min initial denaturation at 95 °C and 35 cycles of 30 s at 95 °C, 45 s at 56 °C and 30 s at 70 °C, followed by a final extension for 8 min at 70 °C. PCR products were purified using NucleoFast 96 PCR plates (Macherey-Nagel) following the manufacturer’s protocol, and eluted in 30 μl water. Both strands of the PCR products were directly sequenced with Applied Biosystems BigDye-Terminator technology on an ABI 3730xl automatic DNA sequencer using the primers from PCR amplifications. Forward and reverse sequences from each directly sequenced amplicon were inspected, manually edited where necessary in Chromas Lite 2.1 (Technelysium Pty Ltd), and assembled in single sequences.

To obtain sequence information from the chloroplast genome we sequenced the *ycf1* gene. PCR amplification and sequencing followed Bartha *et al*.^38^. When due to low DNA quality not the entire gene region could be amplified by a single PCR, we used internal primers to amplify the locus in two separate parts. All primer sequences are provided in Supplementary Table S2. Forward and reverse sequences from each amplicon were inspected, manually edited where necessary in Chromas, and assembled in single sequences.

### Sequence alignments and phylogenetic analyses

ITS and *ycf1* sequences were manually aligned. We assembled four different datasets: (*i*) including all ITS sequences from the 303 individuals available to us (named ITS_303); (*ii*) a second ITS dataset were we restricted the sample design to include one individual per species resulting in 106 sequences (ITS_106); (*iii*) a set of 65 sequences for the chloroplast gene *ycf1*; (*iv*) and a dataset derived from 65 individuals where *ycf1* and ITS sequences could be obtained. To test different models of sequence evolution Paup* 4.0a150^57^ was used. The Bayesian information criterion (BIC) chose the K80 + G model for the ITS datasets with one individual per species (ITS_106), whereas the Akaike information criterion (AICc) resulted in the SYM + G model. For the larger ITS dataset with multiple individuals for most *Hymenostegis* species (ITS_303) the respective models were JC (AICc) and TrNef + G (BIC). For the *ycf1* dataset the model of sequence evolution was inferred as TVM + G (BIC, AICc), while for the ITS sequences, which were combined with the *ycf1* data the models were TrNef + G (AICc) and HKY + G (BIC). In the cases were different models were inferred by AICc and BIC separate phylogenetic analyses were conducted and the trees were compared afterwards.

Potential conflict between the ITS and *ycf1* loci was tested prior to combination by the partition-homogeneity test (ILD; ^58^) in Paup*. This test was implemented with 100 replicates, using a heuristic search option with simple addition of taxa, TBR branch swapping and MaxTrees set to 1000.

Bayesian phylogenetic inference (BI) was conducted in MrBayes 3.2.6^59^. In BI two times four chains were run for 20 million generations for all datasets specifying the respective model of sequence evolution. In all analyses we sampled a tree every 1000 generations. Converging log-likelihoods, potential scale reduction factors for each parameter and inspection of tabulated model parameters in MrBayes suggested that stationary had been reached in all analyses. The first 25% of trees of each run were discarded as burn-in.

For all datasets also parsimony analyses were conducted in Paup* using a two-step heuristic search as described in Blattner^34^ for the ITS_303 dataset, including all 303 individuals, with the searches restricted to a maximum number of 10,000 trees. Heuristic searches with 25 random-addition-sequences were conducted for ITS_106, including one individual per species. Here as well a for *ycf1* and the combined ITS + *ycf1* datasets no maximum tree number restrictions were imposed. To test clade support bootstrap analyses were run on all datasets with re-sampling the large ITS_303 dataset 250 times, all other datasets 500 times with the same settings as before, except that we did not use random-addition sequences. Maximum-likelihood analyses of the ITS and combined ITS + *ycf1* datasets were conducted in RAxML ^60^ using the GTRGAMMA setting and 500 bootstrap re-samples to estimate branch support values.

### Divergence time estimations

To roughly estimate clade ages and divergence times within the analyzed taxa, we used a crown-group age of 12.4 My, given by Wojciechowski^32^, as secondary calibration point for *Astragalus* including *Biserrula pelecinus*. We used Beast 2.4.3^61^ to analyze the dataset of ITS sequences (ITS_106) with K80 + G as model of sequence evolution, a random local clock^62^, and the calibrated Yule prior^63,64^. The node age was defined as a normal-distributed prior (12.4 ± 1.45 My, 95% distribution interval). Monophyly was enforced for the *Astragalus* clade including *Biserrula pelecinus*, as this taxon according to Wojciechowski^32^, Podlech and Zarre^7^ and also the results of our study falls into *Astragalus*. Three independent analyses were run for 20 million generations each, sampling every 1000 generations. Effective sample size (ESS) and convergence of the analyses were assessed using Tracer 1.6 (part of the Beast package). Appropriate burn-ins were estimated from each trace file, discarded and all analyses were combined with LogCombiner (part of the Beast package). A maximum clade credibility (MCC) tree was summarized with TreeAnnotator (part of the Beast package) using the option “Common Ancestor heights” for the nodes.

## Electronic supplementary material

Supplementary information
