# Supplementary material for: Molecular phylogeny and divergence times of Astragalus section Hymenostegis: An analysis of a rapidly diversifying species group in Fabaceae
Source: Sci Rep. 2017 Oct 25;7:14033. doi: 10.1038/s41598-017-14614-3 (PMC5656595; doi:10.1038/s41598-017-14614-3)
Supplement: Supplementary file 1 — Supplementary information [file 41598_2017_14614_MOESM1_ESM.pdf]

Molecular phylogeny and divergence times of *Astragalus* section *Hymenostegis*: An analysis of a currently diversifying species group in Fabaceae

Ali Bagheri[1*], Ali Asghar Maassoumi[2], Mohammad Reza Rahiminejad[1], Jonathan Brassac[3], Frank R. Blattner[3,4]

[1]Department of Biology, Faculty of Sciences, University of Isfahan, Isfahan, 81746-73441, Iran
[2]Botany Research Division, Research Institute of Forests and Rangelands, Agricultural Research, Education and Extension Organization (AREEO), 13185–116, Tehran, Iran
[3]Leibniz Institute of Plant Genetics and Crop Plant Research (IPK), 06466 Gatersleben, Germany
[4]German Centre of Integrative Biodiversity Research (iDiv) Halle-Jena-Leipzig, 04103 Leipzig, Germany
[*]a.bagheri@sci.ui.ac.ir

Supplemental online materials

Table S1 Individuals analyzed.

Sp.	Loc.	Alt.	Herb. No.	Coll.	ITS	*ycf*1
A. altiusculus	Qazvin:Alamut, vicinity of Evan Lake	1600	**97943**	Bagheri	LT622362	-
	Qazvin: Alamut, Moallem Kelayeh	2100	**50998 (Holo)**	Assadi & Maassoumi	LT622361	-
A. anguranensis	Zanjan: Dandi to Aghkand	2300	**98282**	Bagheri	LT622366	-
	Zanjan: Mahneshan to Pari	2300	**98281**	Bagheri	LT622365	-
	Zanjan: Mahneshan, Damirlu Mt.	2600	**100454**	Mahmoodi	LT622367	-
	Zanjan: Mahneshan, Anguran, Belgheis Mt.	2800	**78520 (Holo)**	Maassoumi &Mozaffarian	LT622364	KY071131
	Zanjan: Mahneshan, Anguran	2300	**64809**	Maassoumi	LT622363	-
A. angustispicatus	Mazandaran: Hezar, Abgarm	2250	**33142**	Assadi& Mozaffarian	✓	-
	Tehran: Damavand, Chenar to Daryache Tar	2700	**78017**	Mozaffarian	LT622369	-
A. annularis	Iran	-	**51921**	Maassoumi & Abouhamzeh	AB051912.1	-
A. askius	Iran	-	**6344**	Riazi	AB231113.1	-

Sp.	Loc.	Alt.	No.	Coll.	ITS	*ycf1*
A. assadabadensis	Hamedan: Assadabad	2250	**5009**	Safikhani et al.	KP658436.1	-
	Hamedan: Assadabad	2000	**101441**	Rahiminejad &Bagheri	LT622370	-
	Hamedan: Assadabad	2030	**278 (MSB) (Iso)**	Gharemani-nejad	LT622371	KY071132
	Lorestan: Garrin	-	**s.n.**	Dehshiri	LT622373	-
	Hamedan:Hamadan to Qorveh	2480	**65000 (MSB)**	Mozaffarian	LT622372	-
A. asteris	Iran	-	**30957**	Runemark & Mozaffarian	AB051917.1	-
A._atrokurdicus	Zanjan: Zanjan to Bijar	1550	**6911**	Bagheri	LT622374	-
A. austrokhorasanicu	Gorgan: Shahrud, Tash to Gorgan	2800	**85446**<br>**85454**	Assadi & Hamdi	LT622375<br>LT622376	-<br>KY071133
A.austromahneshanensi s	Zanjan: Mahneshan	2160	**8955 (Holo)**	Bagheri	LT622377	KY071134
	Zanjan: Dandi to Tekab	2300	**98290**	Bagheri	LT622378	-
A.austrotaromenensis	Zanjan: Zanjan to Tarom	2250	**6942**	Bagheri	LT622379	-
A. bashmaghensis	Kurdestan: Saghez, Divandarre, Bashmagh	2300	**70063 (Holo)**	Mozaffarian	LT622380	KY071135

Sp.	Loc.	Alt.	No.	Coll.	ITS	*ycf*1
A. blattneri	East Azarbayejan: Kiamaki Dagh	2040	**55288 (MSB)**	Podlech, Maassoumi &Zarre	LT622381	-
	East Azarbayejan:Tabriz toBostanabad, Saeidabad	2000	**97989**<br>**97990 (Holo)**	Bagheri	LT622382<br>LT622383	-<br>-
A. bounophillus	Tehran: Damavand Mt.	-	**313 (MSB) (Iso)**	Kotschy	LT622385	-
	Mazandaran: Kojur to Baladeh	2650	**98552**<br>**98560**	Bagheri	LT622387<br>LT622388	-<br>-
	Qazvin:Alamut, Moallem Kelayeh	1800	**97947**	Bagheri	LT622386	-
	Mazandaran: Drre Lar	2500	**13423**	Wendelbo & Assadi	LT622384	-
	Mazandaran: Baladeh to Pole Zanguleh	-	**98562**<br>**98563**	Bagheri	LT622389<br>LT622390	-<br>-
A. bradosticus	West Azarbayejan:Uromieh, Mvana, Hakki	2100	**75424**	Mozaffarian, Maassoumi & Safavi	LT622391	-
	West Azarbayejan:Uromieh, Mvana, Hakki	2100	**75442 (Holo)**	Mozaffarian, Maassoumi & Safavi	LT622392	KY071136

Sp.	Loc.	Alt.	No.	Coll.	ITS	*ycf* 1
A. brunsianus	Semnan: Shahmirzad to Fuladmahaleh	2300	**40443**	Assadi& Mozaffarian	LT622395	-
	Teharan: Doshan Tapeh	-	**s.n.** **(B-14)** **(B)** **(Holo)**	Bruns	LT622396	KY071137
	Zanjan: Gheydar, Dehjalal	2140	**101469**	Bagheri	LT622393	-
	Tehran: Bibi Shahbanu Mt.	1350	**15215**	Babakhanlou & Amin	LT622394	-
A. callistachys **(sect. *Microphysa*)**	Esfahan: Golpayegan to Muteh	1850	**76713**	Maassoumi & Mozaffarian	LT622397	KY071138
A. campylanthus **(*Campylanthus*)**	Markazi: Arak, Khan–e Miran, Sefid Khany Mt.	2850	**63774**	Mozaffarian	LT622398	KY071139
A. capax	Tehran: Damavand Mt.	3300	**64734** **(Holo)**	Khatamsaz, Akhiani& Abouhamzeh	LT622399	-
A. cephalanthus **(sect. *Microphysa*)**	Fars: Dasht-e Arjan, Kazeron, Kotal Pirehzan	2400	**17497 (G)**	Foroughi	LT622400	KY071140
A. chehreganii	West Azarbayejan:Salmas, Serrow	1840	**98433**	Maassoumi & Bagheri	LT622404	-
	West Azarbayejan: Serrow to Salmas	1700	**98410**	Rahiminejad & Bagheri	LT622402	-
	West Azarbayejan:Uromieh, Ghoushchi	1550	**98412**	Maassoumi & Bagheri	LT622403	-
	West Azarbayejan:Uromieh, Ghoushchi	1850	**41877** **(MSB)** **(Iso)**	Rechinger	LT622401	KY071141

Sp.	Loc.	Alt.	No.	Coll.	ITS	*ycf1*
A. chrysostachys	Isfahan, (Aderbaydan!!)	**-**	**4401-A (MSB)**	Aucher-Eloy	LT622405	KY071142
	Zanjan: Mahneshan	2200	**6809**	Bagheri	LT622406	-
	West Azarbayejan:Uromieh to Oshnavyeh, Ghasemlo	1500	**98078**	Bagheri	LT622407	-
	Zanjan: Mahneshan to Pari	2300	**98277**	Bagheri	LT622408	-
A. chrysostachys* var *dolichourus	Siwend	-	**1073 (WU) (Holo)**	Stapf	LT622409	-
A. chrysostachys* var *parisiensis	Lorestan: Paris Mt.	-	**15880 (W) (Holo) 1958-7596**	Koelz	LT622410	-
A.chrysostacys* var *khorasanicus	Khorasan: Kopet-Dagh Mt., Alamali	1600	**4802 (4804) (MSB) (Holo)**	Rechinger & Aellen	LT622411	-
A. ciloensis	Turkey, Hakkari, Cilo Mt.	2750	**23974 (M) (Holo)**	Davis & Polunin	LT622412	KY071143
	Turkey: Muradiye	2640	**2765**	Karaman	LT622413	-

Sp.	Loc.	Alt.	No.	Coll.	ITS	*ycf1*
A. conicus	Khorasan: Ghuchan to Dareh Gaz	1820	**21424**	Assadi & Maassoumi	LT622414	-
	Khorasan: Dareh Gaz	1840	**225**	Vafaii	LT622415	-
	Khorasan: Binalud	-	**23**	-	LT622416	KY071144
	Khorasan: Mashhad, Torbat heydarieh	1020	**38234 (MSB)**	Zarre et al.	LT622417	-
					LT622418	-
	Khorasan: Shirvan	2400	**41375 (W)**	Termeh, Moussavi		
	Khorasan: Quchan	1600	**53723 (M)**	Rechinger	LT622419	-
	Khorasan: Ghuchan, Bajgiran	1600	**6314 (W)**	Schmid	LT622420	-
	Semnan: Semnan to Firuzkuh	2000	**97903**	Bagheri	LT622421	-
A. dandianus	Zanjan: Dandi to Tekab	2300	**98283**	Bagheri	LT622422	KY071145
A. demonstratus	Zanjan: Mahneshan to Pari	2000	**64804 (Holo)**	Maassoumi	LT622423	KY071146
A. depressus	Germany	-	**39745**	Podlech	AB231147.1	JQ801556.1
	Italy	-	**- (M)**	Dietrish		
A. dianat nejadii	East Azarbayejan: Ahar to Kaleybar, Arasbaran	1550	**69611 (Holo)**	Gharemani-Nejad& Zarre	LT622426	KY071147
	East Azarbayejan:Arasbaran	1800	**55329 (MSB)**	Podlech, Maassoumi & Zarre	LT622425	-
	East Azarbayejan:Arasbaran	1750	**20195**	Assadi & Maassoumi	LT622424	-

Sp.	Loc.	Alt.	No.	Coll.	ITS	*ycf1*
A. doghrunensis	East Azarbayejan: Arasbaran, Doghrun Mt.	2800	**23974 (Holo)**	Assadi & Sardabi	LT622428	-
	East Azarbayejan:Arasbaran, Doghrun Mt.	2500	**23921**	Assadi & Sardabi	LT622427	-
	East Azarbayejan:Arasbaran	2550	**70269**	Jamzad et al.	LT622429	-
A. epiglottis	Morocco	-	**45851**	Podlech	AB051910.1	-
A. expetitus	West Azarbayejan: Tekab	2400	**12223 (Holo)**	Wendelbo, Assadi & Shirdelpur	LT622430	KY071148
	Zanjan: Dandi to Tekab	2300	**98291**	Bagheri	LT622432	-
	Zanjan: Dandi to Tekab	2300	**98228**	Bagheri	LT622431	-
***A. fasciculifolius* (sect. *Poterium*)**	Fars: Nurabad,Babamonir	960	**63414**	Mozaffarian	LT622433	KY071149
A. ferruminatus	Lorestan: to Ilam, Shahhabad	1510	**24706 (Holo)**	Seraj	LT622435	KY071150
	Kermanshah: Dalahoo, Kerend	2100	**1396**	Hamzehee & Hatami	LT622434	-
	Kermanshah: Eslamabade Gharb	1600	**39509**	Zarre et al.	LT622436	-
***A. glaucacanthos* (*Poterion*)**	Tehran: E. of Tehran	1350	**15102**	Dini & Arazm	LT622437	-
A. glumaceus	Zanjan: Dandi to Tekab	2300	**98284**	Bagheri	LT622439	-
	Zanjan: Mahneshan to Pari	2300	**98280**	Bagheri	LT622438	KY071151

Sp.	Loc.	Alt.	No.	Coll.	ITS	*ycf1*
A. gueruenensis	Turkey:Sivas, Gurun	1700	**15070 (MSB) (Holo)**	Huber-Morath	LT622440	-
	Turkey: Gurun	1700	**2777**	Karaman	LT622441	-
	Turkey, Erzurum	1800	**43485 (MSB)**	Nydegger	LT622442	-
	Turkey: Sivas	1500	**4415 (B)**	Hein	LT622443	-
A. hakkianus	West Azarbayejan:Uromieh, Silvana, Haki	2100	**98057**<br>**98059**	Bagheri	LT622444<br>LT622445	KY071152<br>-
A. hirticalyx	Armenia: Syunik	1900	**08-0565 (M)**	Vitek et al	LT622446	-
	East Azarbayejan:Arasbaran, Makidi	2400	**20216**	Assadi & Maassoumi	LT622447	-
A. hymenocystis	West Azarbayejan:Uromieh, Ghoushchi	1550	**98413**	Maassoumi & Bagheri	LT622454	KY071154
	West Azarbayejan:Salmas to Uromieh, Ghoushchi	1650	**57000**	Maassoumi & Abouhamzeh	LT622452	-
	West Azarbayejan:Uromieh, Ghoushchi	1700	**80287**	Maassoumi & Nikchehreh	LT622453	-
	Turkey: Van	2700	**53881 (W)**	Rechinger	LT622451	-
	Turkey: Van	-	**2383**	Mehmet	LT622450	-

Sp.	Loc.	Alt.	No.	Coll.	ITS	*ycf1*
A. dejectus	Zanjan: Gheydar, Gheydar Mt.	2490	**9084**<br>**9079**<br>**(Holo)**	Bagheri	LT622449<br>LT622448	-<br>KY071153
A. hymenostegis	West Azarbayejan:Salmas, Serrow	1840	**98428**	Maassoumi & Bagheri	LT622457	-
	West Azarbayejan:Uromieh, Salmas to Pereshkhoran	2450	**98072**	Bagheri	LT622456	KY071155
	West Azarbayejan: Seyed Khajeh	-	**491**<br>**(MSB)**<br>**(Iso)**	Szovits	LT622455	-
A. kapherrianus	Zanjan: Tarom	2200	**6145**<br>**9036**<br>**9053**	Bagheri	LT622460<br>LT622461<br>LT622462	KY071156<br>-<br>-
	Zanjan: Badamestan	2000	**41107**<br>**(MSB)**	Lamond & Iranshahr	LT622459	-
	Zanjan , Gilvan	2350	**41076-E**<br>**(MSB)**	Moussavi, Habibi & Tehrani	LT622458	-
A. karl heinzii	Ardabil: Masuleh to Khalkhal	2100	**86477**<br>**(Holo)**	Assadi	KT997422.1	-
	Zanjan: Gheydar, Gheydar Mt.	2300	**9**<br>**6617**<br>**98260**<br>**98274**	Bagheri	KT997425.1<br>KT997424.1<br>KT997426.1<br>KT997423.1	-<br>-<br>-<br>-

Sp.	Loc.	Alt.	No.	Coll.	ITS	*ycf1*
A. kauzarashensis	West Azarbayejan:Salmas, Serrow	1840	**101436**	Maassoumi & Bagheri	LT622463	-
	West Azarbayejan:Uromieh, Salmas to Kozarash	2250	**98062**	Bagheri	LT622464	-
	West Azarbayejan:Uromieh, Salmas to Pereshkhoran	2450	**98076**	Bagheri	LT622465	-
A. kohrudicus	Alborz: Karaj	1500	**16030**	Foroughi	✓	-
	Tehran: Tehran, Sorkhehesar	1400	**19018 (W)**	Amin & Bazargan	KT894776.1	KY071157
	Tehran: Tehran, Takestan	1450	**36618**	Assadi & Mozzafarian	KT894777.1	-
	Ghazvin: Saudash-Bulagh	1300	**6874 (B) (Holo)**	Bornmüller	KT894779.1	-
	Sorkhe Hesar, Haraz road	1530	**12468 (MSB)**	Foroughi et al.	✓	-
	Tehran: Ghazvin, Takestan	-	**49783 (W.N. 12247)**	Termeh	KT894778.1	-

Sp.	Loc.	Alt.	No.	Coll.	ITS	*ycf1*
A. lagopodioides	Turkey: Agri, Hasiran	2650	**140 (MSB)**	Engel	LT622466	-
	West Azarbayejan:Uromieh, Salmas to Pereshkhoran	2450	**98075**	Bagheri	LT622472	KY071158
	West Azarbayejan:Khoy, Ghotour, Razi	2170	**98393**	Maassoumi & Bagheri	LT622473	-
	Turkey: Van	1900	**2589**<br>**2590**	Karaman	LT622467<br>LT622468	-<br>-
	Turkey: Van	2550	**2626**	Karaman	LT622469	-
	Turkey: Van	2500	**728 (M)**	Rix et. al.	LT622470	-
	Turkey: Gevas	2200	**787-93-11 (MSB)**	Ehrendorfer Sorger	LT622471	-
A. lagopoides	East Azarbayejan:Tabriz, Kandovan, Sahand Mt.	2850	**98042**	Bagheri	LT622474	-
	East Azarbayejan:Sofian to Shabestar, Kamar sefid	2300	**98305**	Maassoumi & Bagheri	LT622475	-
A. laguriformis	Kermanshah: Bistun	1800	**1096**	Hamzehee & Hatami	KP658435.1	-
	Iraq: Sefin Mt.	1000	**1194 (B)(Iso)**	Bornmüller	KP658433.1	KY071159
A. laxispicatus	Hamedan: Boghati Mt.	2000	**280 (W) W.N. 2009-15399**	Ghahreman inejad	LT622483	-
	Hamedan:Hamedan to Avaj	2100	**97914**	Bagheri	LT622484	-

Sp.	Loc.	Alt.	No.	Coll.	ITS	*ycf1*
A. lagurus	Turkey: Bitlis	2400	**109 (MSB)**	Engel	LT622476	-
	Turkey: Ozlap	2100	**2761**	Karaman	LT622477	-
	Caucasus	-	**276 (B)**	-	LT622478	-
	Turkey: Van	1800	**2802**	Mehmet	LT622479	-
	Turkey: Van	-	**5505**	Mehmet	LT622480	-
	East Azarbayejan:Jolfa, Zonouz	2300	**98321**	Maassoumi & Bagheri	LT622481	-
	West Azarbayejan:Khoy, Ghotour, Razi	2170	**98395**	Maassoumi & Bagheri	LT622482	-
A. leptynticus	Semnan: Firuzkuh	2450	**13014 (Holo)**	Wendelbo & Foroughi	LT622485	KY071160
	Semnan: Shahmirzad to Foladmahaleh	2300	**97906**	Bagheri	LT622487	-
	Semnan: Semnan to Firuzkuh	2400	**97909**	Bagheri	LT622488	-
	Zanjan: Zanjan to Dandi, Morassa village	2250	**99842**	Mahmoodi	LT622489	-
	Zanjan: Dandi to Tekab	2300	**9005**	Bagheri	LT622486	-
A. leucargyreus	Hamedan: Elwend Mt.	-	**s.n. (B-13) (B) (Holo)**	Strauss	LT622492	-
	Hamedan: Elwend Mt.	3100	**4501**	Ariavand	LT622490	-
	Hamedan: Tuyserkan, Elvend Mt.	3300	**95790**	Dehshiri& Mostafavi	LT622491	KY071161

Sp.	Loc.	Alt.	No.	Coll.	ITS	*ycf* 1
A. makuensis	West Azarbayejan: Maku, Chaldoran to Khoy	2150	**98385**<br>**98387**	Maassoumi & Bagheri	LT622493<br>LT622494	KY071162<br>-
A. marivanensis	Kurdestan:Sardasht to Mahabad, Marivan	1400	**80658**	Maassoumi &Shahsavari	LT622497	-
	Kurdestan:Baneh to Marivan	1650	**29326 (Holo)**	Runemark, Mozaffarian	LT622496	KY071163
	Iraq: Sefin Mt.	1200	**1177 (B)**	Bornmüller	LT622495	-
	Kurdestan	-	**98445**	Maassoumi & Bagheri	LT622498	-
A. melanostictus	West Azarbayejan:Oshnavyeh to Uromieh, Gnjabad	1900	**101438**	Bagheri	LT622499	-
	Khorasan: Ghuchan, Alamali neck	2000	**10375**	Mousavi & Pariyab	LT622501	-
	Zanjan: Mianeh	1800	**6758**	Bagheri	LT622506	-
	Kurdestan: Sanandaj, Salavatabad	2200	**101442**	Bagheri	LT622500	-
	West Azarbayejan: Rezaeiye	1600	**36816 (W)** **1979- 00978**	Mosavi & Tehrani	LT622504	-
	Hamadan: Malayer, Ekbatan Dam	-	**52092**	Maassoumi	LT622505	KY071164
	Soltanabad	-	**s.n. (145) (B) (Iso)**	Strauss	LT622503	-
	Iraq: Sefin Mt.	1200	**1177-b (B)**	Bornmüller	LT622502	-
	Zanjan: Mahneshan	2100	**6784**	Bagheri	LT622507	-
	Zanjan: Gheydar, Karasf	-	**8991**	Bagheri	LT622508	-

Sp.	Loc.	Alt.	No.	Coll.	ITS	*ycf1*
A. mesopotamicus	Mesopotamia	-	**1275 (MSB) (Holo)**	Aucher-Eloy	LT622509	-
A. montis nacarouzii	Kurdestan:Saghez to Baneh, Pir omran, Nacarouz Mt.	2500	**7219 (Holo)**	Maroofi & Moradi	LT622510	KY071165
A. montis sarali	Kurdestan: Sanandaj, Chehelcheshme Mt., Saral	2350	**74926**	Mozaffarian, Maassoumi & Safavi	LT622511	-
A. mostafa-assadii	Zanjan: Abhar to Gheydar	2300	**101444**<br>**101447**<br>**s.n.**	Bagheri	LT622512<br>LT622514<br>LT622518	-<br>-<br>-
	Zanjan: Abhar to Gheydar	-	**101445**	Bagheri	LT622513	-
	Zanjan: Abhar to Gheydar	1940	**6598**<br>**6628**	Bagheri	LT622515<br>LT622516	-<br>-
	Zanjan: Zanjan to Bijar	2070	**6884**	Bagheri	LT622517	-
***A. murinus* (*Anthylloiedei*)**	Chaharmahal: Dena,from bideh,bijan pass	3000	**76750**	Maassoumi & Mozaffarian	LT622519	KY071166
A. naftabensis	Mazandaran: Baladeh to Pole Zanguleh	3110	**98566**<br>**98567**	Bagheri	LT622522<br>LT622523	-<br>-
	Mazandaran: Nur, Naftab	3200	**6465 (B) (Holo)**	Rechinger	LT622521	-
	Mazazndaran: Nur, Elika	3000	**6410 (B) (Holo)**	Rechinger &Manucheri	LT622520	-

Sp.	Loc.	Alt.	No.	Coll.	ITS	*ycf*1
A. nervistipulus	Kurdestan: Sanandaj to Kamyaran	2030	**86020**	Maassoumi& Safavi	LT622527	-
	Kurdestan: Kamyaran to Sanandaj	1450	**35264 (B)**	Zarre et al.	LT622525	-
	Siwend	-	**333 (Holo) (B)**	Stapf	LT622524	-
	Kurdestan: Sanandaj to Marivan	1800	**42914 (B)**	Rechinger	LT622526	-
A. nirensis	Ardabil: Ardabil to Nir	2250	**97976**<br>**97977**	Bagheri	LT622528<br>LT622529	KY071167<br>-
A. nowjianensis	Lorestan: Nozhian	-	**12088**	Gholmrezaei	LT622530	-
A. paralurges	Zanjan: Gheydar, Gheydar Mt.	2300	**7032**	Bagheri	LT622534	-
	Zanjan: Gheydar, Karasf Mt.	1930	**6646**	Bagheri	LT622533	-
	Zanjan: Abhar to Gheydar	2300	**101448**<br>**101449**	Bagheri	LT622531<br>LT622532	-<br>KY071168
A. paralurgiformis	Zanjan: Abhar to Gheydar	2300	**101446**	Bagheri	LT622535	-
	Zanjan: Dehjalal	2060	**6855**	Bagheri	LT622536	-
	Zanjan: Gheydar, Gheydar Mt.	2300	**8829**	Bagheri	LT622537	-

Sp.	Loc.	Alt.	No.	Coll.	ITS	*ycf1*
A. pauxillis	Zanjan: Mahneshan to Dandi, Anguran, Belgheis Mt.	2200	**64818 (Holo)**	Maassoumi	LT622538	KY071169
	Zanjan: Soltanieh, Arjin, Sheikhsari	2100	**60604**	Ranjbar, Gharemani-Nejad, Zarre	LT622539	-
	Zanjan: Gheydar, Gheydar Mt.	2100	**9072**	Bagheri	LT622540	-
A. pediculariformis	Zanjan: Soltanieh, Arjin, Sheikhsari	2250	**69603 (Holo)**	Ranjbar, Ghahremani & Zarre	LT622543	KY071170
	Zanjan: Gheydar, Gheydar Mt.	2300	**14**<br>**9800**	Bagheri	LT622541<br>LT622545	-<br>-
	Zanjan: Gheydar, Gheydar Mt.	-	**6858**	Bagheri	LT622542	-
	Zanjan: Gheydar, Yengi kand	2055	**8858**	Bagheri	LT622544	-
A. persicus	Arak: Arak to Malayer	2100	**48080**	Mozzafarian Mohammadi	LT622555	KY071171
	Lorestan: Borujerd	2200	**1268 (B)**	Køie	LT622554	-
A. pluriflorus	Zanjan: Mahneshan to Pari	2300	**6812a (Holo)**<br>**6812b**	Bagheri	LT622556<br>LT622557	-<br>-
A. pseudoparaluges	Zanjan: Gheydar, Yengi kand	2050	**8865**	Bagheri	LT622558	-

Sp.	Loc.	Alt.	No.	Coll.	ITS	*ycf1*
A. pereshkhoranicus	West Azarbayejan: Silvana, Mavana, Bardarash	3000	**69902 (69602) (Holo)**	Mozaffarian	LT622550	-
	West Azarbayejan: Silvana, Mavana	1500	**101437**	Rahiminejad &Bagheri	LT622546	-
	West Azarbayejan: Uromiyeh, Targavar	2100	**69605**	Ranjbar, Gharemani-Nejad, Zarre	LT622549	-
	West Azarbayejan: Silvana to Salmas	1600	**85185**	Assadi	LT622551	-
	West Azarbayejan: Razhan, Khalil khuh	3200	**48846-a (B) (Holo)**	Rechinger	LT622548	-
	West Azarbayejan: Khalil kuh	2400	**48659 (B)**	Rechinger	LT622547	-
	West Azarbayejan:Salmas, Serrow	1840	**98430**	Maassoumi & Bagheri	LT622553	-
	West Azarbayejan: Maku, Chaldoran to Gharakhach	2300	**98362**	Maassoumi & Bagheri	LT622552	-
A. pseudopersicus	East Azarbayejan: Tabriz to Sperkhan	1600	**69853 (Holo)**	Mozzafarian	LT622560	KY071172
	East Azarbayejan: Sperkhan, Sahand Mt.	2850	**37535 (MSB)**	Mozzafarian Mohammadi	LT622559	-
A. purpureocalycinus	Turkey: Van	-	**s.n.**	Mehmet	LT622563	-
	Turkey: Gurpinar	2200	**2603**	Karaman	LT622561	-
	Turkey: Van	2000	**4880**	Altan	LT622562	-

Sp.	Loc.	Alt.	No.	Coll.	ITS	*ycf1*
A. qorvehensis	Hamedan: Qorveh, Veynesar	1900	**82602 (MSB) (Holo)**	Maassoumi & Safavi	LT622565	KY071173
	Hamedan:Hamedan to Ghorveh, Veynesar	2000	**97928**	Bagheri	LT622566	-
	Zanjan: Soltanieh	1850	**40920 (W)**	Termeh & Moussavi	LT622564	-
A. qoturensis	West Azarbayejan: Khani ziarat, Habashi, Ghotur	3000	**49644 (M) (Holo)**	Rechinger & Renz	LT622568	KY071174
	West Azarbayejan: Ghotur, Abgarm	1900	**7330**	Amini	LT622569	-
	Turkey: Van	2750	**2653**	Karaman	LT622567	-
A. qeydarnabiensis	Zanjan: Gheydar, Gheydar Mt.	2330	**8801 (Holo)**	Bagheri	LT622570	KY071175
	Zanjan: Gheydar, Gheydar Mt.	2300	**98253**	Bagheri	LT622571	-
A. recognitus	East Azarbayejan:Tabriz toBostanabad, Tikmedash	1900	**97997**	Bagheri	LT622574	
	Ardebil: Meresht to Arpachai	2500	**34304**	Mozaffarian & Nowrozi	LT622572	KY071176
	East Azarbayejan: Arpa Chai	2400	**55369 (MSB)**	Podlech et al.	LT622573	
A. remotispicatus	Zanjan: Gheydar, Zarand, Zarand Mt.	2300	**97932a (Holo) 97932b**	Bagheri	KT997427.1 KT997428.1	- -
A. rijabensis	Kermanshah: Eslam abad, Kerend	-	**101773 (Holo)**	Mozafarian	LT622575	-

Sp.	Loc.	Alt.	No.	Coll.	ITS	*ycf1*
A. rubriflorus	Mazandaran	1900	**75446**	-	LT622577	KY071177
	Mazandaran: Kandovan	3000	**69623B**	Ghahremani-Nejad	LT622576	-
	Mazandaran: Janat Rudbar	3100	**78446**	Maassoumi	LT622578	-
A. rubrostriatus	Zanjan: Gheydar, Gheydar Mt.	2300	**98252**<br>**7114**	Bagheri	LT622585<br>LT622584	-<br>-
	Zanjan: Abhar to Gheydar	2000	**101443**	Bagheri	LT622580	KY071178
	Alborz: Gasawand Mt.	-	**s.n. (B-12) (B) (Holo)**	Strauss	LT622579	-
	Zanjan: Sohrein	2080	**6873**	Bagheri	LT622582	-
	Tehran: Damavand	1700	**21256 (MSB)**	Attar & Okhovvat	LT622581	-
	Zanjan: Zanjan to Tarom	2250	**6844**	Bagheri	LT622583	-
A. salavatabadensis	Kurdestan: Salavatabad	2300	**48473 (M) (Holo)**	Rechinger	LT622589	KY071179
	Hamedan:Hamedan to Ghorveh, Veynesar	2000	**101440**	Bagheri	LT622586	-
	Kurdestan: Sanandaj	2000	**42753 (MSB)**	Rechinger	LT622587	-
	Kurdestan: Salvatabad	2300	**42811 (B)**	Rechinger	LT622588	-
	Kurdestan: Sanandaj	2000	**84854**	-	LT622590	KY071180

Sp.	Loc.	Alt.	No.	Coll.	ITS	*ycf*1
A. sciureus	Qazvin:Alamut, Moallem Kelayeh, Ghostinlar neck<br>Alborz: Karaj<br>Alborz: Elborz Mt.	1800<br>2000<br>-	**97946**<br>**19326**<br>**520 (MSB) (Iso)**	Bagheri<br>Amin & Bazargan<br>Kotschy	KT894781.1<br>KT894780.1<br>KT894782.1	KY071181<br>-<br>-
A. seidabadensis	East Azarbayejan:Sarab to Nir, Bozghosh Mt.<br>East Azarbayejan:Sarab to Nir, Soltanabad, Bozghosh Mt.<br>East Azarbayejan:Bostanabad, Saeidabad<br>East Azarbayejan:Tabriz, Seidabbad	2500<br>2150<br>1800<br>-	**98014**<br>**98018**<br>**98011**<br>**98295**<br>**s.n. (MSB) (Iso)**	Bagheri<br>Bagheri<br>Maassoumi & Bagheri<br>Bunge & Bienert	LT622592<br>LT622593<br>LT622591<br>LT622594<br>LT622595	-<br>-<br>-<br>KY071182<br>-
A. simakanensis	Fars: Bavanat<br>Chaharmahal va Bakhtiari: Vardanjan	2500<br>2300	**s.n. (Holo)**<br>**98204**	Teyebi Khorrami<br>Bagheri	LT622597<br>LT622596	KY071183<br>-
A. sohrevardianus	Zanjan: Gheydar, Karasf to Sohrevard	2120	**8846**	Bagheri	LT622598	**-**
A. sphericus	West Azarbayejan:Uromieh, Salmas to Kozarash	2250	**98061**	Bagheri	LT622603	-

Sp.	Loc.	Alt.	No.	Coll.	ITS	*ycf1*
A. sosnowskyi	West Azarbayejan:Chaldoran to Khoy, Makhmur	2050	**98391**	Maassoumi & Bagheri	LT622601	-
	West Azarbayejan: Maku	1860	**82567**	Maassoumi & Safavi	LT622600	KY071184
	Turkey:Erzurum	2000	**46632 (MSB)**	Nydegger	LT622599	-
	Georgia: Tana	-	**s.n. (MSB) (Iso)**	Kikodzes	LT622602	-
A. straussii	Hamedan:Hamedan to Avaj	2200	**97917**<br>**97918**	Bagheri	LT622606<br>LT622607	KY071185<br>-
	Hamedan:Hamedan to Ghahavand	2350	**64468**	Mozaffarian	LT622604	-
	Hamedan:Hamadan to Saveh	1900	**65029 (MSB)**	Mozaffarian	LT622605	-
A. subkohrudicus	Zanjan: Gheydar, Gheydar Mt.	2090	**8812 (Holo)**	Bagheri	LT622608	KY071186
A. sublaguriformis	Zanjan: Zanjan to Mahneshan	2120	**8952**	Bagheri	LT622609	-
	Zanjan: Zanjan to Mahneshan	2120	**8953 (Holo)**	Bagheri	LT622610	-
***A. submitis* (*Anthyloiedi*)**	Ghazvin: Evan, Khashe chal	3100	**75886**	Jamzad et al.	LT622611	KY071187
A. subrecognitus	Zanjan: Mahneshan to Pari	2300	**8960**<br>**98279**	Bagheri	LT622615<br>LT622616	KY071188<br>-
	Zanjan: Mahneshan to Pari	2300	**6808**<br>**6811**	Bagheri	LT622613<br>LT622614	-<br>-
	Zanjan: Mahneshan, Damirlo Mt.	2739	**100334-B**	Mahmoodi	LT622612	-

Sp.	Loc.	Alt.	No.	Coll.	ITS	*ycf1*
A. tabrizianus	East Azarbayejan:Sofian to Shabestar, Kamar sefid	1570	**98302**	Maassoumi & Bagheri	LT622622	KY071189
	East Azarbayejan:Tabriz, Kandovan, Sahand Mt.	2850	**98044**	Bagheri	LT622619	-
	East Azarbayejan:Bostanabad to Tabriz, Shibli neck	2100	**98050**	Bagheri	LT622620	-
	East Azarbayejan:Bostanabad to Tabriz, Saeid abad	-	**69614**	Ranjbar, Ghahremani-Nejad& Zarre	LT622618	-
	West Azarbayejan: Bukan to Mahabad	1650	**101439**	Bagheri	LT622617	-
	East Azarbayejan:Sofian to Shabestar, Kamar sefid	2300	**98301**	Maassoumi & Bagheri	LT622621	-
***A. tricholobus* (*Campylanthus*)**	Zanjan: Zanjan to Dandi, Kaltakeh village	1900	**99583**	Mahmodi	LT622623	-
A. trifoliastrum	Turkey: Gurpinar	2150	**2650**	Karaman	LT622624	-
	Turkey: Van	1850	**44283 (W) 1985- 10183**	Davis	KP658434.1	KY071190
A. uraniolimneus	East Azarbayejan:Jolfa, Zonouz	2300	**98350**	Maassoumi & Bagheri	LT622627	KY071191
	West Azarbayjan: Silvana, Mavana	2800	**69901**	Mozaffarian	LT622626	-
	Caucasus: Pambakaski	2200	**s.n. (W) W. N. 1983-10642**	Valak	LT622625	-

Sp.	Loc.	Alt.	No.	Coll.	ITS	*ycf1*
A. vaginans	Turkey: Van<br>Turkey: Goksun	-<br>540	**4432**<br>**42588 (MSB)**	Mehmet<br>Nydegger	LT622629<br>LT622628	-<br>KY071192
A. velenovskyi	East Azarbayejan: Kandovan, Sahand Mt.	2900	**18283 (W)**	Grant	LT622630	-
A. vernaculus	Isfahan: Najafabad to Khorramabad<br>Isfahan: Chadegan to Tiran	-<br>2430	**1010 (MSB) (Holo)**<br>**98205**<br>**98207**	Manuchehri<br>Bagheri	LT622631<br>LT622632<br>LT622633	KY071193<br>-<br>-
A. woronowii	Turkey: Batum<br>West Azarbayejan: Salmas, Pereshkhoran<br>East Azarbayejan:Jolfa, Zonouz	2200<br>2100<br>2300	**5946 (B) (Holo)**<br>**69939**<br>**98331**	Woronow<br>Mozaffarian<br>Maassoumi & Bagheri	LT622634<br>LT622635<br>LT622636	-<br>-<br>-
A. zohrabi	West Azarbayejan:Maku to Chaldoran<br>Turkey: Armenia prope Baibut	1750<br>-	**98367**<br>**69 (MSB) (Iso)**	Maassoumi & Bagheri<br>Bourgeau	LT622638<br>LT622637	-<br>-
Biserrula pelecinus	Australia	-	-	-	AB287409.1	-
Oxytropis kotschyana	Ghazvin: Moalem kelaye	1900	**98085**	Bagheri	LT622640	-

Sp.	Loc.	Alt.	No.	Coll.	ITS	*ycf1*
Oxytropis karjaginii	Zanjan: Zanjan to Dandi, Talkhab village	2300	**99612**	Mahmoodi	LT622639	-
Oxytropis pilosa	Romania	-	**662398 (without herbarium name)**	Bartha	-	JQ801550.1
	USSR	-	-	-	AF121759.1	-

Table S2 PCR primers used for the amplification of the *ycf1* region of the chloroplast genome in *Astragalus* sect. *Hymnostegis* and graphical representation of their position in the gene. Relative primer positions are given in the graph below.

Primer name	Primer sequence (5'–3')	Reference
ycf1-F	ATCMATGGACAARTTGGTT	Bartha et al. 2012 (64)
ycf1-R	CTAATCGATAATTTGGCC	Bartha et al. 2012 (64)
IntF1	AAAGGAGCAAACGAAGAAGC	Bartha et al. 2012 (64)
IntR	TCGTTGAGGTAGTTATTTCG	Bartha et al. 2012 (64)
ycf1_Int_SF	ATATCTATCTCGCTTAAACC	this study
ycf1_Int_SRb	TGGTACAAGCCAAGGATTC	this study
ycf1_int_SRc	CCTTTAGATCGTTGAGGTAG	this study

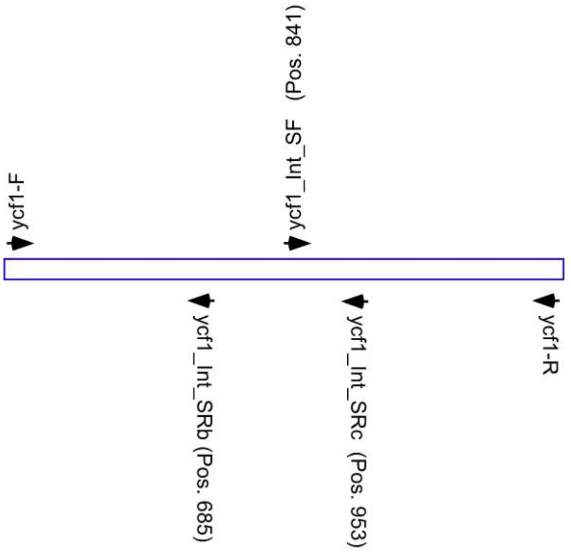

Table S3 Results of age estimations in a BEAST analysis. Taxon group refers to the tree in Figure S3.

Taxon group	Node support Yule	Dates Yule (in My)		Calibration point
		**mean age**	**95% HPD**	
hymeno	0.96	3.55	2.13-5.08	
angus	1	0.96	0.06-2.19	
brado	1	0.72	0.12-1.46	
chehr	0.97	1.42	0.45-2.51	
laxi	0.97	0.26	0-0.78	
atro	0.9	0.61	0.05-1.34	
austro	0.91	0.99	0.3-1.81	
vagi	0.8	2.31	1.18-3.59	
dianat	1	1	0.3-1.96	
hymeno+outgroup1	1	3.96	2.41-5.59	
callis	1	0.25	0.01-0.6	
glauc+callis	0.99	1.58	0.67-2.64	
fasc+leuc	0.98	1.74	0.7-2.85	
outgroup1	0.99	2.64	1.44-4.02	
hymeno+outgroup1+aste	1	5.29	3.33-7.34	
annu+epi	1	2.97	1.52-4.61	
biser+annu	1	5.55	3.32-7.91	
Oxytropis	1	1.27	0.25-2.62	
Astragalus + Biserrula				calibrated–Normal (12.4. ±1.45)

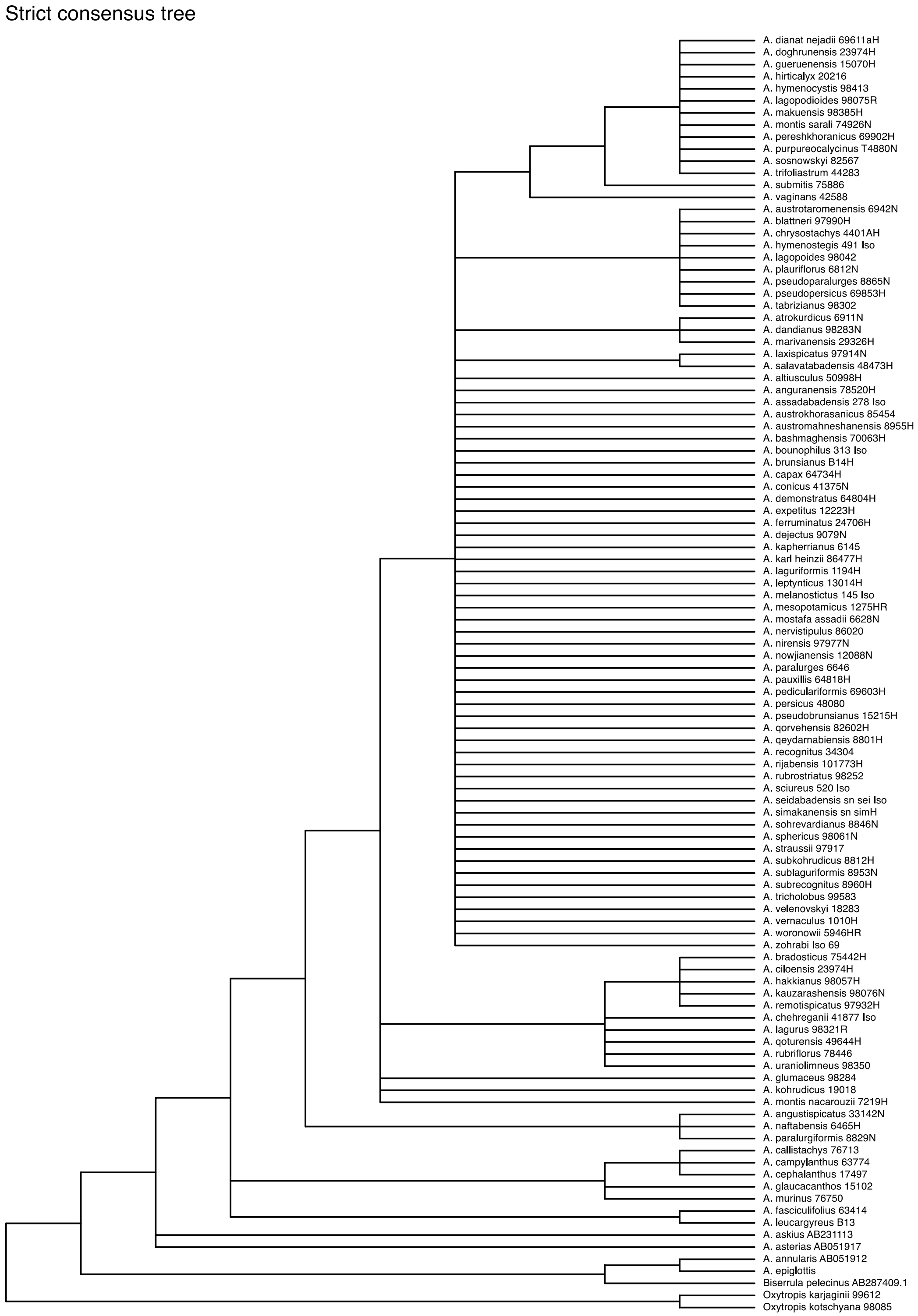

Figure S1. Strict consensus of 359 maximum parsimony trees derived from nrDNA ITS sequences of a dataset including one individual each per sect. *Hymenostegis* species.

Figure S2. Phylogenetic tree derived from a maximum-likelihood reconstruction in RAxML (GTRGAMMA) based on a nrDNA ITS dataset of 106 *Astragalus* and outgroup individuals. Numbers along branches give bootstrap support values ≥50%.

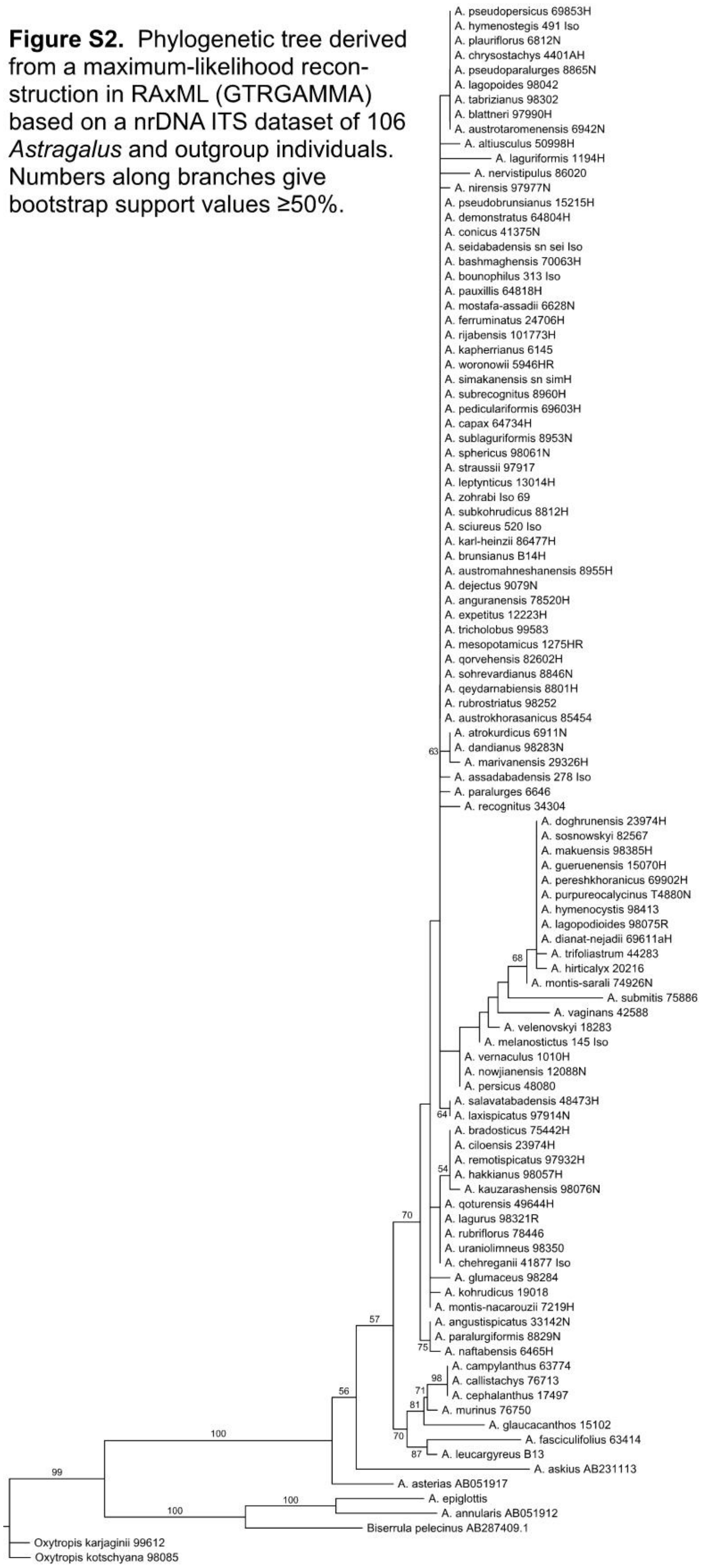

Figure S3. Phylogenetic tree derived from Bayesian inferrence of a nrDNA ITS dataset of 303 *Astragalus* and outgroup individuals. Numbers along branches give posterior probabilities. Asterisks behind species names indicate new species names currently in the process of valid publication, ° depicts three species formerly not included in sect. *Hymenostegis*.

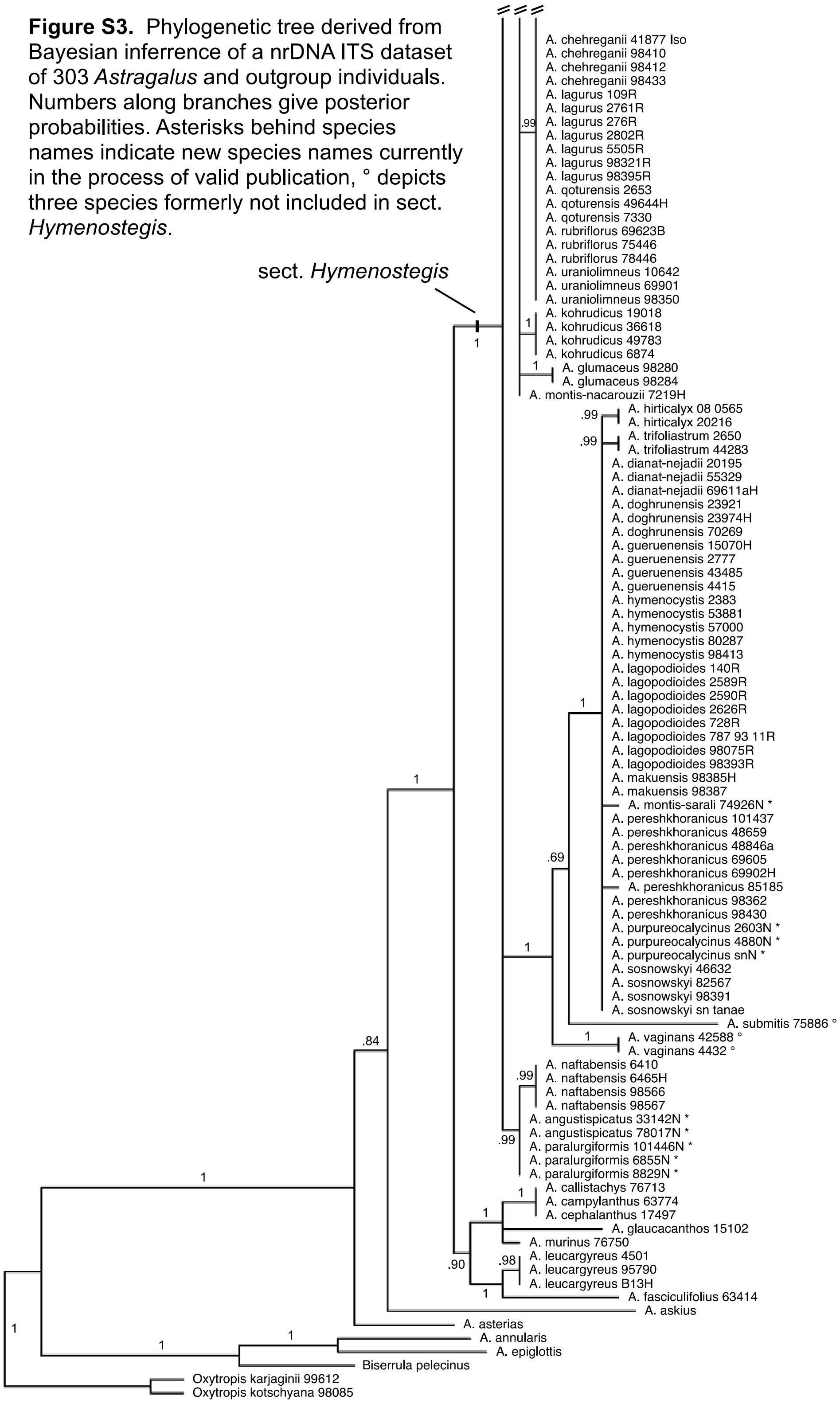

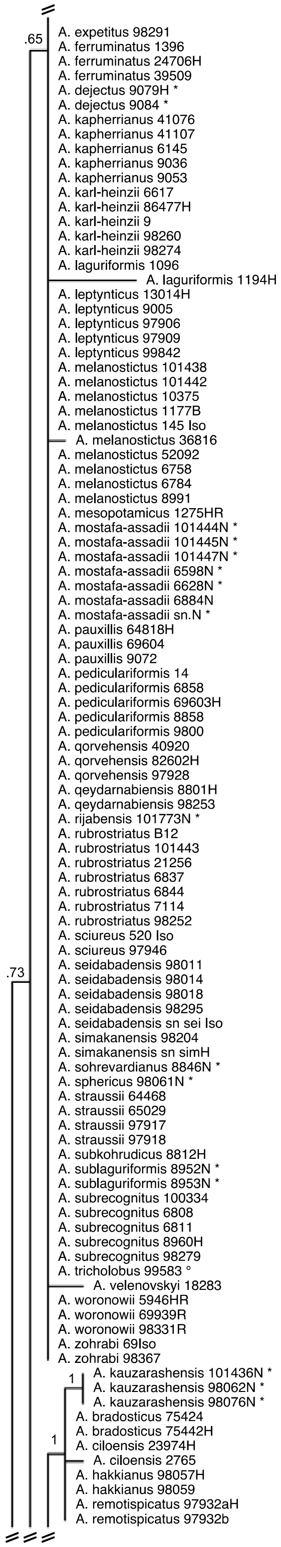

Figure S3. Continued.

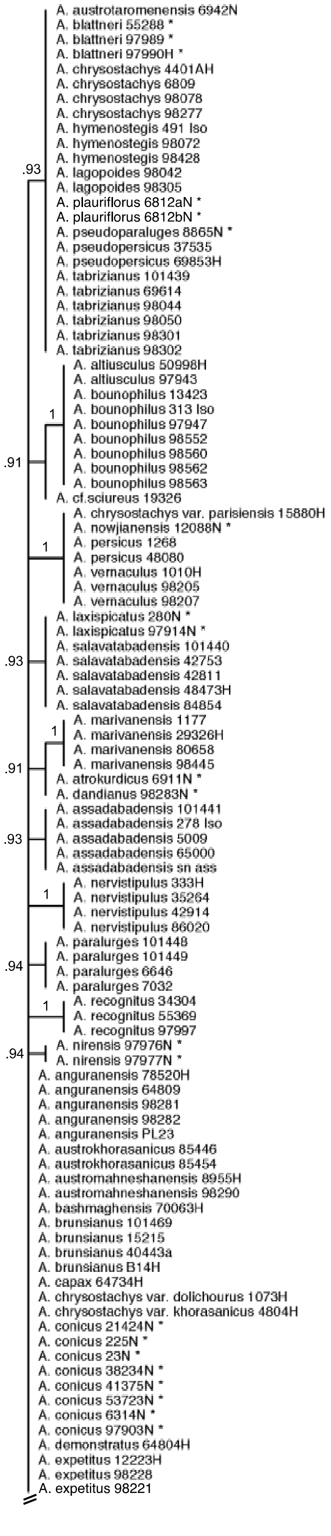

Figure S3. Continued.

Figure S4. Maximum-likelihood tree derived from the combined sequences of ITS and *ycf1*. Numbers along branches provide support values derived from 500 bootstrap re-samples.

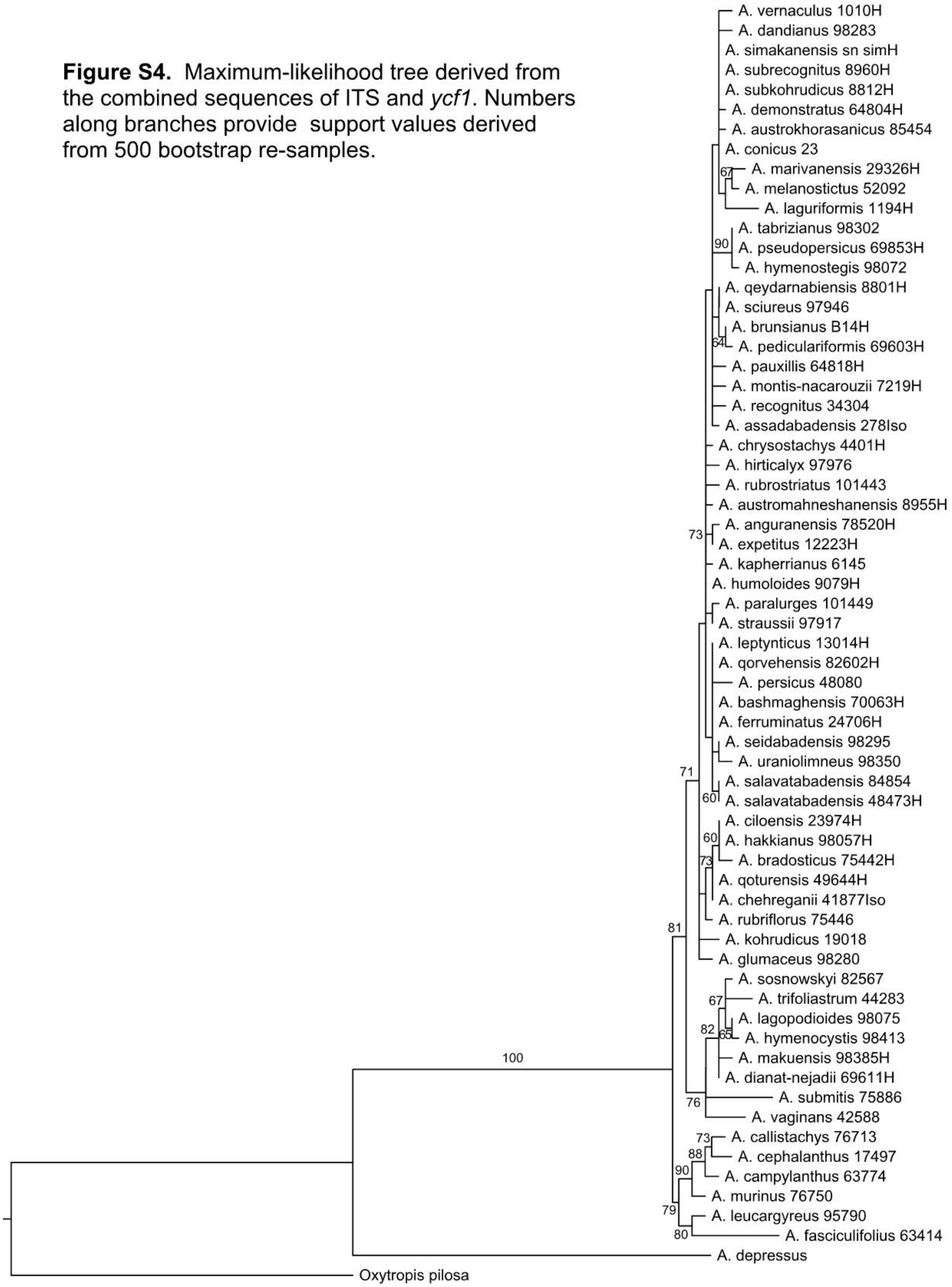

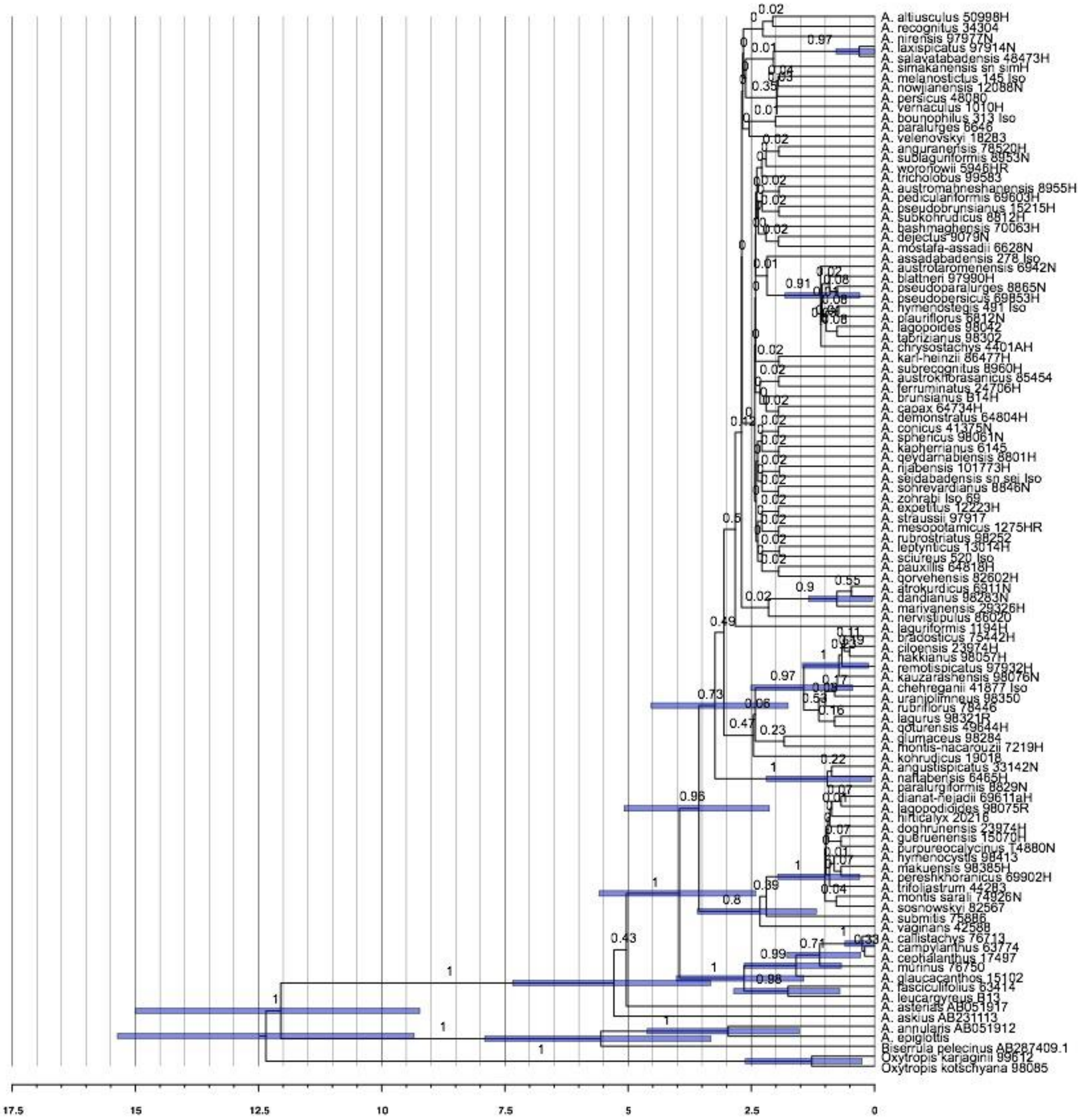

Figure S5. Dated phylogeny calculated from nrDNA ITS sequences using the calibrated Yule prior and a secondary calibration on the crown group of *Astragalus* (including *Biserrula pelecinus*) and *Oxytropis* as a normal-distributed prior (12.4 ± 1.45 My). Numbers along branches indicate Bayesian posterior probabilities >0.70. Node bars indicate 95% highest probability density intervals (HPD) for the ages. The scale at the bottom provides a timeline in million years before present.
